# Non‐vitamin K antagonist oral anticoagulants versus vitamin K antagonists in atrial fibrillation patients with previous stroke or intracranial hemorrhage: A systematic review and meta‐analysis of observational studies

**DOI:** 10.1002/clc.23647

**Published:** 2021-05-20

**Authors:** Zongwen Guo, Xiaoli Ding, Zi Ye, Weiling Chen, Yijian Chen

**Affiliations:** ^1^ Department of Critical Care Medicine Soochow University Suzhou Jiangsu Province China; ^2^ Clinical Laboratory The First Affiliated Hospital of Gannan Medical University Ganzhou Jiangxi Province China; ^3^ Internal Medicine Royal North Shore Hospital St Leonards New South Wales Australia; ^4^ Department of Hematopathology Gannan Medical University Ganzhou Jiangxi Province China

**Keywords:** anticoagulants, atrial fibrillation, intracranial hemorrhage, stroke

## Abstract

Several observational studies have compared the effectiveness and safety outcomes between nonvitamin K antagonist oral anticoagulants (NOACs) and vitamin K antagonists (VKAs) in atrial fibrillation (AF) patients with a history of either stroke/transient ischemic attack (TIA) or intracranial hemorrhage. Therefore, our current meta‐analysis aimed to address this issue. The Cochrane Library, PubMed, and Embase databases were systematically searched until December 2020 for all relevant observational studies. We applied a random‐effects model to pool adjusted hazard ratios (HRs) and 95% confidence intervals (CIs) for this meta‐analysis. A total of 10 studies were included. Among patients with a history of stroke/TIA, the use of NOACs versus VKAs was associated with decreased risks of stroke (HR, 0.82, 95% CI 0.69–0.97), systemic embolism (HR, 0.73, 95% CI 0.61–0.87), all‐cause death (HR, 0.87, 95% CI 0.81–0.94), major bleeding (HR, 0.77, 95% CI 0.64–0.92) and intracranial hemorrhage (HR, 0.54, 95% CI 0.38–0.77). Among patients with a history of intracranial hemorrhage, the use of NOACs versus VKAs was associated with reduced risks of stroke (HR, 0.81, 95% CI 0.68–0.95), all‐cause death (HR, 0.68, 95% CI 0.49–0.94), and intracranial hemorrhage (HR, 0.66, 95% CI 0.51–0.84). Compared with VKAs, the use of NOACs exhibited superior efficacy and safety outcomes in AF patients with previous stroke/TIA, and the use of NOACs was associated with reduced risks of stroke, all‐cause death, and intracranial hemorrhage in patients with a history of intracranial hemorrhage.

## 1 INTRODUCTION

Oral anticoagulants (OACs) have been extensively prescribed for patients with atrial fibrillation (AF) as the first‐line treatment to prevent stroke or other systemic embolisms.^1^, ^2^ Vitamin K antagonists (VKAs), predominantly warfarin, were the only available class of oral anticoagulants over the past decades until 2009 when nonvitamin K antagonist oral anticoagulants (NOACs, including dabigatran, apixaban, rivaroxaban, and edoxaban) were introduced.^3^ Several studies have been conducted to compare the effectiveness and safety of NOACs versus VKAs among patients with AF.

Although it has been well‐demonstrated by previously published meta‐analyses that NOACs have an advantageous risk–benefit profile in stroke prophylaxis, intracranial hemorrhage, and all‐cause mortality in comparison with warfarin among patients with AF,^3^, ^4^, ^5^, ^6^ the heterogeneity of the effectiveness and safety between NOACs and VKAs has not been revealed for a specific subgroup of patients, namely AF patients with a past medical history of stroke/transient ischemic attack (TIA), or intracranial hemorrhage. Given that patients in this subgroup are susceptible to recurrent cerebrovascular ischemic events^7^ as well as intracranial hemorrhage,^8^ which is the most lethal adverse effect of anticoagulants,^9^ it is thus of significance to scrutinize their different responses to NOAC and VKA anticoagulant therapies. In this meta‐analysis of real‐world studies, we address the discrepancies in effectiveness and safety outcomes between NOACs and VKAs in AF patients who have previously been diagnosed with a stroke/TIA, or intracranial hemorrhage.

## 2 METHODS

Our current meta‐analysis was performed based on the Cochrane handbook for systematic reviews. The results of this study were arranged based on the Preferred Reporting Items for Reporting Systematic Reviews and Meta‐analyses (PRISMA). Given that no patients were involved in the establishment of the research question, the outcome measures, and the design or the implementation of this meta‐analysis, ethical approval was necessary. The data, methods, and materials of this study are available to others for purposes of reproducing the results or replicating procedures by contacting the corresponding author.

### 2.1 Search strategy

The three electronic databases (the Cochrane Library, PubMed, and Embase) were systematically searched up to December 2020 by two reviewers. We potentially included studies that evaluated the comparisons of effectiveness and safety between NOACs and VKAs in AF patients with a history of stroke and/or intracranial hemorrhage. We used the following search items including: (1) atrial fibrillation; AND (2) non‐vitamin K antagonist oral anticoagulants OR direct oral anticoagulants OR dabigatran OR rivaroxaban OR apixaban OR edoxaban; AND (3) vitamin K antagonists OR warfarin OR coumadin OR acenocoumarol OR phenprocoumon OR fluindione OR phenindione OR anisindione. Cross‐referencing was also applied to identify potentially missed studies. The reference lists of meta‐analyses and systematic reviews were reviewed and retrieved for more studies. We applied no restrictions on language in the searches.

### 2.2 Study eligibility

Eligible studies were included if they met all of the following criteria: (1) observational studies focusing on nonvalvular AF patients with a history of stroke/TIA or patients with a history of intracranial hemorrhage; (2) comparisons of outcomes between NOACs (dabigatran, rivaroxaban, apixaban, or edoxaban) and VKAs (warfarin, coumadin, phenprocoumon, acenocoumarol, fluindione, phenindione, or anisindione); (3) the effectiveness outcomes included stroke, systemic embolism, and all‐cause death, while the safety outcomes included major bleeding, intracranial hemorrhage, and gastrointestinal bleeding. We only included studies that reported the effect estimates: adjusted hazard ratios (HRs) and 95% confidence intervals (CIs). If one study reported adjusted HRs in multiple models, the most adjusted model was included.

We applied the following exclusion criteria: (1) studies were duplicated publications; (2) publications had no relevant data (e.g., reviews, case reports, editorials, and comments); (3) studies focused on AF patients after interventions, such as cardioversion, ablation, or left‐atrial appendage closure.

### 2.3 Study selection and data collection

The retrieved pieces of literature were imported into the NoteExpress V3.0 software (Beijing Aegean Sea Music Technology Co., Ltd.; http://www.inoteexpress.com/aegean/), and we deleted duplicate records. Two reviewers independently screened the titles and abstracts of the retrieved records. Then, the full texts of the records were reviewed to identify all potentially eligible studies. Any disagreement was addressed by discussion or was resolved by an additional reviewer. For each included study, we mainly collected the following data: the first author and publication year, study design, data source, inclusion period, type of NOACs and VKAs, the reported effectiveness and safety outcomes, and the adjusted effect estimates. Two reviewers independently collected the data and compared the results to ensure coherence.

### 2.4 Quality assessment

For the observational studies, two reviewers independently evaluated the methodological quality using the Newcastle‐Ottawa Scale (NOS) tool. This tool involved three blocks: the selection of cohorts, the comparability of cohorts, and outcome evaluation. A study can be awarded a maximum of one point for each numbered item within the selection and outcome categories. A maximum of two points can be given for comparability. A study with an NOS score of <6 was defined as low quality.^10^, ^11^ Disputable issues were resolved by consensus, or discrepancies were resolved by an additional reviewer.

### 2.5 Statistical analysis

The statistical analyses were performed using Review Manager 5.3 software (the Nordic Cochrane Center, Rigshospitalet, Denmark). We used heterogeneity analysis to quantify the degree of heterogeneity of I^2^ calculations. I^2^ > 50% of the value indicated substantial heterogeneity. The natural logarithms of HRs and standard errors of the included studies were calculated.^12^ Considering the possible heterogeneity existing in the eligible studies, we applied a random‐effects model with an inverse variance method for this meta‐analysis. To verify the robustness of the results, sensitivity analyses were performed by excluding the studies one by one or using a fixed‐effects model. Subgroup analyses could not be performed due to the limited data. According to the Cochrane handbook, the funnel plot should generally not be considered when the included studies were less than 10. The comparisons were considered two‐sided, and p < .05 was considered statistically significant.

## 3 RESULTS

### 3.1 Included study features

The flow diagram of the literature search is shown in Supplemental Figure 1. A total of 4685 studies were identified during the electronic search, and we deleted 1624 duplicate publications among the the Cochrane Library, PubMed, and Embase. A total of 3006 studies were excluded based on title and abstract screenings. Then, 55 studies were screened based on the full texts. Finally, a total of 10 observational studies were potentially in this meta‐analysis.^13^, ^14^, ^15^, ^16^, ^17^, ^18^, ^19^, ^20^, ^21^, ^22^ Six^13^, ^14^, ^15^, ^16^, ^18^, ^19^ and three studies^20^, ^21^, ^22^ focused on AF patients with a history of stroke/TIA, and a history of intracranial hemorrhage, respectively, and one study assessed these two populations.^17^ The baseline characteristics of the included studies are shown in Table 1 and Supplemental Tables 1–2. Regarding the quality assessment, the 10 observational studies exhibited acceptable quality.

**TABLE 1:** Baseline characteristics of the included studies

Included studies	Data source	Study period	Age (y)	Females (%)	HAS‐BLED (mean)	CHA2DS2‐VASc (mean)	AF population	Number of participants	NOACs	VKAs	Outcomes of interest	Follow‐up (years)	Quality assessment^a^
Yang L‐2020	Medicare Part D	January 2013–December 2014	76.42 ± 8.66	56.10	3.64 ± 0.94	4.54 ± 1.76	Stroke/TIA	4927	DA; RIV; API	Warfarin	IS, SE, GIB	0.66	7 points
Seiffge DJ‐2019^14^	7 European and Japanese prospective, observational cohorts	NA	77.33 ± 9.65	48.00	3.00 ± 1.48	5.00 ± 1.48	IS	4912	NOACs	Phenprocoumon, warfarin	IS, ICH, death	At least 0.25	9 points
Xian Y‐2019^15^	Patient‐Centered Research into Outcomes Stroke Patients Prefer and Effectiveness Research study; Get with The Guidelines‐Stroke program	October 2011–December 2014	80.00 ± 8.91	56.30	NA	4.00 ± 1.48	IS	11 662	NOACs	Warfarin	Death, IS, SE, ICH, GIB	3.12	8 points
Larsen TB‐2014^16^	Danish national prescriptionregistry	August 2011–May 2013 (DA); August 2009–May 2013 (warfarin)	76.77 ± 9.26	47.47	2.93 ± 0.94	4.73 ± 1.33	Stroke/TIA	6141	DA	Warfarin	IS, TIA, IS/TIA	1.04	8 points
Komen JJ‐2019^17^	Stockholm Healthcare Database	September 2011–June 2018	73.61 ± 10.96	44.30	NA	3.29 ± 1.92	IS; ICH	4967	NOACs	Warfarin	Death	2.00	7 points
Park J‐2019^18^	National Health Insurance Service of Korea	January 2010–April 2018	75.20 ± 9.10	49.00	4.20 ± 1.10	5.90 ± 1.40	Stroke	61 568	DA; RIV; API; EDO	Warfarin	Stroke, MB, death	0.70	8 points
Coleman CI‐2017^19^	US Truven MarketScan data	January 2012–June 2015	72.72 ± 13.03	53.07	3.94 ± 0.82	5.00 ± 1.48	Stroke/TIA	9684	DA; RIV; API	Warfarin	IS, ICH, MB	0.55	7 points
Nielsen PB‐2019^20^	Danish nationwide databases	January 2003–April 2017	77.4 ± 9.0	42.80	NA	4.30 ± 1.60	ICH	622	NOACs	Warfarin	IS, ICH	3.00	8 points
Tsai C‐2020^21^	Taiwan National Health Insurance Research Database	January 2012–December 2016	76.3 ± 10.2	45.5	4.31 ± 1.15	5.43 ± 1.81	ICH	4540	NOACs	Warfarin	IS, ICH, MB, death	5.00	8 points
Lee S‐2020^22^	Korean Health Insurance Review and Assessment database	January 2010–April 2018	73.7 ± 9.6	44.2	4.40 ± 1.20	4.00 ± 1.50	ICH	5712	NOACs	Warfarin	IS,ICH	9.27	8 points

*Note*: Hypertension, age ≥ 75 years, diabetes mellitus, prior stroke/transient ischemic attack (2 points); CHA2DS2‐VASc, congestive heart failure/left ventricular ejection fraction ≤40%, hypertension, age ≥ 75 years (2 points), diabetes mellitus, prior stroke/transient ischemic attack/thromboembolism (2 points), vascular disease, age 65–74 years, female sex.

Abbreviations: API, apixaban; CHADS2, congestive heart failure; DA, dabigatran; EDO, edoxaban; GIB, gastrointestinal bleeding; HAS‐BLED, Hypertension, Abnormal liver/renal function, Stroke, Bleeding history or predisposition, Labile international normalized ratio, Elderly, Drugs/alcohol concomitantly; ICH, intracranial hemorrhage; IS, ischemic stroke; NA, not available; NOACs, non‐Vitamin K antagonist oral anticoagulants; RIV, rivaroxaban; SE, systemic embolism; TIA, transient ischaemic attack; VKAs, vitamin K antagonists.

### 3.2 NOACs versus VKAs in patients with previous stroke/TIA

**FIGURE 1:**
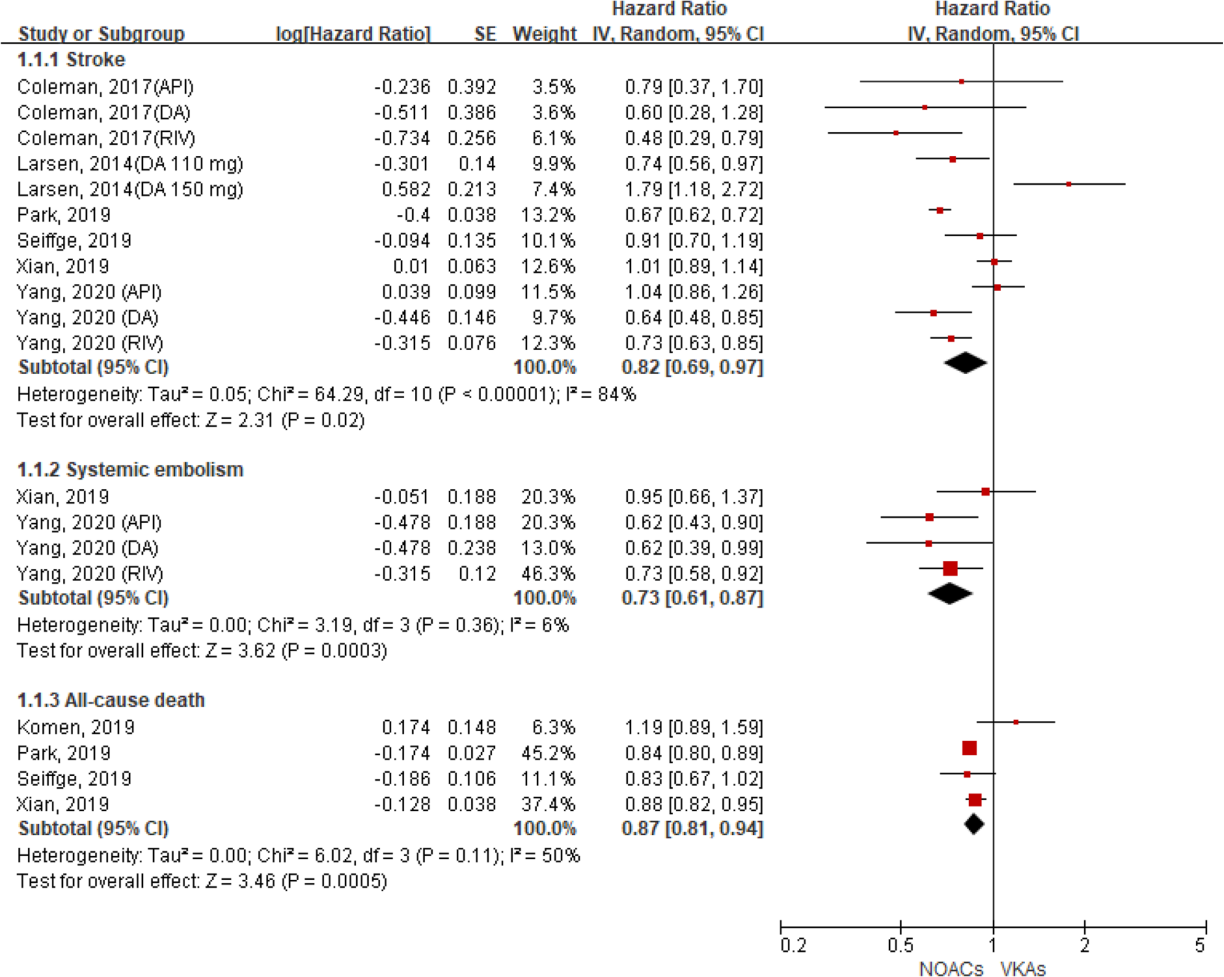
Comparing efficacy of NOACs with VKAs in AF patients with a history of stroke or TIA. AF, atrial fibrillation; API, apixaban; NOACs, non‐vitamin K antagonist oral anticoagulants; CI, confidence interval; DA, dabigatran; IV, inverse of the variance; RIV, rivaroxaban; SE, standard error; TIA, transient ischemic attack; VKAs, vitamin K antagonists

**FIGURE 2:**
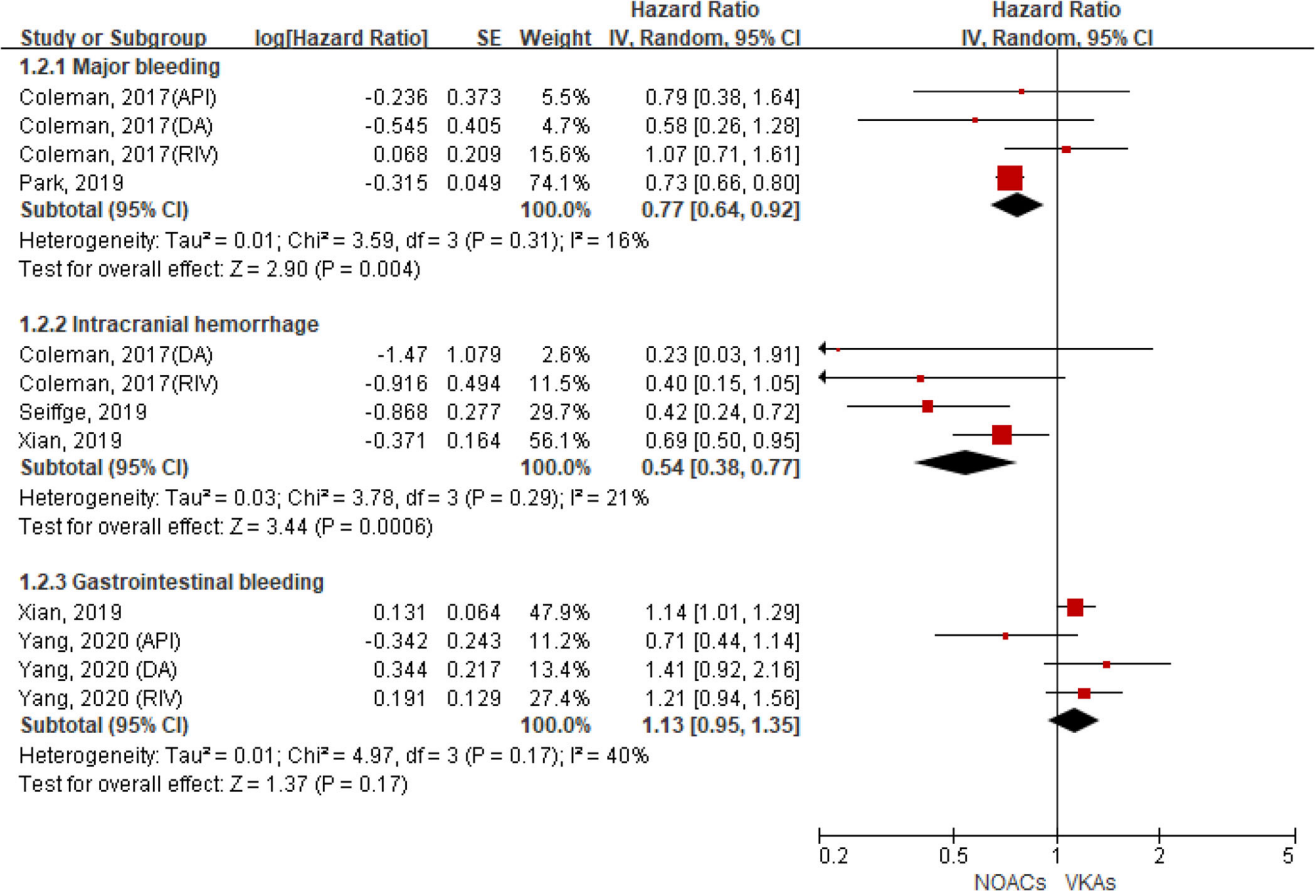
Comparing safety of NOACs with VKAs in AF patients with a history of stroke or TIA. AF, atrial fibrillation; API, apixaban; NOACs, non‐vitamin K antagonist oral anticoagulants; CI, confidence interval; DA, dabigatran; IV, inverse of the variance; RIV, rivaroxaban; SE, standard error; TIA, transient ischemic attack; VKAs, vitamin K antagonists

Seven included studies reported AF patients with a history of stroke/TIA.^13^, ^14^, ^15^, ^16^, ^17^, ^18^, ^19^ Regarding effectiveness outcomes, compared with VKA use, the use of NOACs was associated with reduced risks of stroke (HR, 0.82, 95% CI 0.69–0.97; p = .02; I^2^ = 84%), systemic embolism (HR, 0.73, 95% CI 0.61–0.87; p = .0003; I^2^ = 6%), and all‐cause death (HR, 0.87, 95% CI 0.81–0.94; p = .0005; I^2^ = 50%) (Figure 1). For the safety outcomes, compared with VKA use, the use of NOACs was associated with decreased risks of major bleeding (HR, 0.77, 95% CI 0.64–0.92; p = .004; I^2^ = 16%) and intracranial hemorrhage (HR, 0.54, 95% CI 0.38–0.77; p = .0006; I^2^ = 21%). There was no significant difference in the rate of gastrointestinal bleeding (HR, 1.13, 95% CI 0.95–1.35; p = .17; I^2^ = 40%) (Figure 2).

### 3.3 NOACs versus VKAs in patients with previous intracranial hemorrhage

**FIGURE 3:**
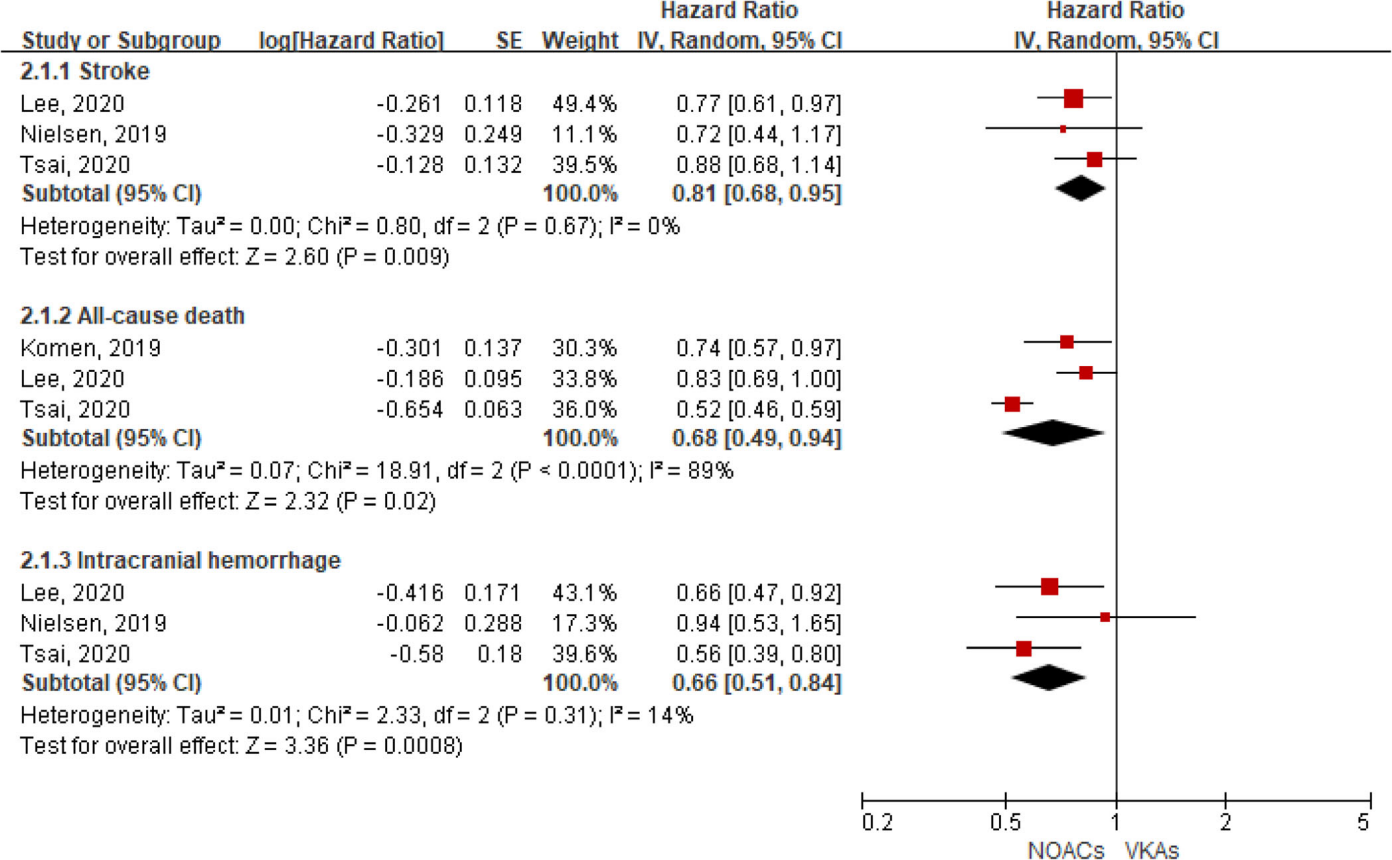
Comparing efficacy and safety of NOACs with VKAs in AF patients with a history of intracranial hemorrhage. AF, atrial fibrillation; API, apixaban; NOACs, non‐vitamin K antagonist oral anticoagulants; CI, confidence interval; DA, dabigatran; IV, inverse of the variance; RIV, rivaroxaban; SE, standard error; TIA, transient ischemic attack; VKAs, vitamin K antagonists

A total of four studies evaluated the comparisons of effectiveness and safety between NOACs and VKAs in AF patients with a history of intracranial hemorrhage.^17^, ^20^, ^21^, ^22^ As shown in Figure 3, compared with VKA use, the use of NOACs was associated with reduced risks of stroke (HR, 0.81, 95% CI 0.68–0.95; p = .009; I^2^ = 0%), all‐cause death (HR, 0.68, 95% CI 0.49–0.94; p = .02; I^2^ = 89%), and intracranial hemorrhage (HR, 0.66, 95% CI 0.51–0.84; p = .0008; I^2^ = 14%). Only 1 study by Tsai et al reported the outcome of major bleeding between NOACs and VKAs (HR, 0.65, 95% CI 0.53–0.67), whereas none of the included studies focused on the outcomes of systemic embolism and gastrointestinal bleeding. The results of this section should be interpreted cautiously due to the limited number of studies included in the analysis.

### 3.4 Sensitivity analysis

After we excluded one study at a time, the corresponding results of this meta‐analysis were stable. In addition, the corresponding results did not change substantially when we reperformed the analyses by using a fixed‐effects model.

## 4 DISCUSSION

To compare the effectiveness and safety outcomes between NOACs and VKAs, our meta‐analysis pooled the data from seven^13^, ^14^, ^15^, ^16^, ^17^, ^18^, ^19^ and four^17^, ^20^, ^21^, ^22^ observational studies for AF patients with stroke/TIA and intracranial hemorrhage, respectively. For patients with histories of stroke/TIA, NOACs illustrated superior effectiveness and safety outcomes compared with VKAs, with statistically significant reductions in stroke or systemic embolic events, all‐cause mortality, major bleeding, and intracranial hemorrhage. Moreover, NOACs showed lower risks of stroke, all‐cause mortality, and intracranial hemorrhage than VKAs for patients with previous intracranial hemorrhage. In general, NOACs were associated with a favorable effectiveness profile for stroke and all‐cause mortality and superior safety outcomes of intracranial hemorrhage compared with VKAs for AF patients with a history of stroke/TIA or intracranial hemorrhage.

The superior effectiveness and safety outcomes of NOACs in stroke and intracranial hemorrhage shown in our results are consistent with similar RCTs and observational studies for AF patients with prior stroke/TIA or intracranial hemorrhage.^11^ Previous studies have elaborated that this superiority is predominantly attributed to significant prevention against hemorrhagic stroke. Given that hemorrhagic stroke is included in both stroke and intracranial hemorrhage, NOACs consequently reduce their risk profiles by halving the risk of hemorrhagic stroke.^3^ As the most lethal complication of anticoagulant treatment, intracranial hemorrhage is a well‐recognized factor in risk–benefit assessment for ischaemic stroke prophylaxis among patients with AF.^8^, ^23^ Anticoagulant treatment accounts for one in six hospital admissions for intracranial hemorrhage, resulting in up to 40% 30‐day mortality.^9^, ^20^, ^24^ Our study revealed a significantly lower incidence of intracranial hemorrhage in patients on NOACs compared with patients on VKAs for both stroke/TIA and intracranial hemorrhage subgroups. This finding is consistent with evidence from RCTs for patients with AF.^3^ However, several observational studies illustrated that compared with VKAs, NOACs had lower or similar rates of intracranial hemorrhage in AF patients with a history of stroke/TIA or intracranial hemorrhage,^25^, ^26^, ^27^, ^28^, ^29^, ^30^ suggesting that NOACs are at least noninferior to VKAs regarding the outcome of intracranial hemorrhage. Due to the limited number of included studies, further investigation is necessary to reveal the discrepancies in intracranial hemorrhage risk profiles between AF patients with and without prior stroke/TIA or intracranial hemorrhage.

The reintroduction of OACs for ischemic event prophylaxis in patients with AF sustaining intracranial hemorrhage should balance the recurrent bleeding risk if the risk of stroke is left untreated.^31^ There was a universal exclusion of patients with a previous intracranial hemorrhage for all four landmark NOAC trials.^32^, ^33^, ^34^, ^35^ Therefore, the present study included all available observational studies comparing the effectiveness or safety between NOACs and VKAs for this underrepresented AF patients sustaining intracranial hemorrhage. To the best of our knowledge, this was the first meta‐analysis emphasizing real‐world data that were lacking in RCTs and providing complementary information to the existing evidence regarding anticoagulation treatment for stroke prophylaxis.

In addition to the associations of NOACs being associated with lower incidences of stroke and intracranial hemorrhage, the all‐cause mortality correlated with NOAC use was significantly reduced compared with that correlated with VKA use. Nevertheless, reductions in all‐cause mortality were not indicated in most Phase 3 NOAC trials,^32^, ^33^, ^35^ except for apixaban^34^ and low‐dose edoxaban.^35^ Our meta‐analysis provides more robust estimates to detect differences in secondary outcomes and subgroups.

### 4.1 Limitations

Some limitations have been identified in our study. First, our statistical analysis was performed without individual participant data for all the included observational studies. Although each study's methodological quality was evaluated by using the NOS tool, we pooled the data from these studies, of which the quality and robustness were unavoidably variable and inconsistent. Second, the severity, imaging, and functional disabilities of prior stroke/TIA or intracranial hemorrhage were not addressed and adjusted, which might have confounded the study outcomes. Third, the protocol of the systematic review and meta‐analysis was not registered in the PROSPERO database. Fourth, although the adjusted data from the included studies were used in our pooled analysis, the residual confounders still existed due to the nature of the observations studies. Fourth, the time in the therapeutic range of warfarin users was not considered in our pooled analysis due to the limited data. As such, we should interpret the results of this meta‐analysis cautiously, and our findings might not be completely generalizable to all the patients in real‐world settings. Finally, only one study by Tsai et al. reported the outcome of major bleeding between NOACs and VKAs, whereas none of the included studies focused on the outcomes of systemic embolism and gastrointestinal bleeding. Further studies should examine these outcomes between NOACs and VKAs in AF patients with a history of intracranial hemorrhage.

## 5 CONCLUSION

Compared with VKAs, the use of NOACs exhibited superior efficacy and safety outcomes in AF patients with a history of stroke/TIA, and the use of NOACs was associated with reduced risks of stroke, all‐cause death, and intracranial hemorrhage in patients with a history of intracranial hemorrhage.

## CONFLICT OF INTEREST

All authors declare that they have no potential conflicts of interest that might be relevant to the contents of this review.

## AUTHOR CONTRIBUTIONS

Data curation: Zongwen Guo, Xiaoli Ding. Formal analysis: Zongwen Guo, Xiaoli Ding. Investigation: Zongwen Guo,Weiling Chen. Methodology: Zongwen Guo, Xiaoli Ding. Software: Zi Ye. Supervision: Yijian Chen. Validation: Zongwen Guo. Writing‐original draft: Zongwen Guo, Xiaoli Ding, Zi Ye. Writing‐review and editing: Yijian Chen.

## Supporting information


**Appendix** S1: Supporting Information

## Data Availability

Availability of data and materials have been described in the manuscript.They are freely available to any scientist who wishes to use them without breaching participant confidentiality.
