# Supplementary material for: Non‐vitamin K antagonist oral anticoagulants versus vitamin K antagonists in atrial fibrillation patients with previous stroke or intracranial hemorrhage: A systematic review and meta‐analysis of observational studies
Source: Clin Cardiol. 2021 May 20;44(7):917–24. doi: 10.1002/clc.23647 (PMC8259149; doi:10.1002/clc.23647)
Supplement: Supplementary file 1 — Appendix S1: Supporting Information [file CLC-44-917-s001.docx]

**Supplemental Table 1. Comorbidities and medications of the included studies**

|  | **Comorbid conditions(%)** | | | | | | | | **Medications(%)** | | | | | |
| --- | --- | --- | --- | --- | --- | --- | --- | --- | --- | --- | --- | --- | --- | --- |
| **Included studies** | **Hypertension** | **Diabetes mellitus** | **Heart failure** | **Ischemic heart disease** | **Myocardial infarction** | **Chronic kidney disease** | **History of stroke or TIA** | **History of bleeding** | **Antiplatelet drugs** | **Beta-Blockers** | **Statins** | **ACEI or ARBs** | **Diuretics** | **Proton-pump inhibitors** |
| Yang L-2020 | 91.08 | 46.15 | 50.82 | NA | 8.64 | 37.48 | 23.17 | 16.59 | 11.96 | NA | NA | NA | NA | NA |
| Seiffge DJ-2019 | 75.07 | 24.66 | NA | NA | NA | NA | 3.52 | NA | 38.60 | NA | 25.88 | NA | NA | NA |
| Xian Y-2019 | 81.03 | 25.78 | 12.73 | NA | 31.12 | NA | 8.09 | NA | 54.54 | NA | NA | NA | NA | NA |
| Larsen TB-2014 | 35.79 | 16.39 | 10.39 | NA | 18.27 | NA | 35.52 | 18.38 | NA | NA | NA | NA | NA | NA |
| Komen JJ-2019 | 65.10 | 16.80 | 16.30 | NA | 5.50 | 6.30 | 19.90 | 9.70 | 44.80 | 52.40 | 28.40 | 38.20 | 20.60 | NA |
| Park J-2019 | 89.75 | 32.98 | 43.39 | NA | 5.94 | NA | 100.00 | 20.63 | 44.56 | NA | NA | NA | NA | 34.32 |
| Coleman CI-2017 | 78.05 | 32.76 | 18.52 | NA | NA | 9.92 | 100.00 | 4.79 | 34.52 | 57.78 | 58.36 | 54.58 | 31.49 | NA |
| Nielsen PB-2019 | 62.10 | 51.60 | 32.60 | NA | 11.60 | 11.40 | 100.00 | NA | NA | NA | NA | NA | NA | NA |
| Tsai C-2020 | 94.10 | 50.15 | 54.96 | NA | 10.40 | 25.00 | 74.74 | 100.00 | 11.20 | NA | NA | NA | NA | NA |
| Lee S-2020 | 90.10 | 32.20 | 41.80 | NA | 5.40 | 16.80 | 20.30 | NA | 34.20 | NA | NA | NA | NA | 30.90 |

ACEIs=angiotensin-converting enzyme inhibitors; ARBs=angiotensin receptor blockers; NA=not available**Supplemental Table 2. Dose of NOACs and TT of warfarin users in the included studies**

| **Included studies** | **Dose of NOACs** | **TTR of warfarin users (%)** |
| --- | --- | --- |
| Yang L-2020 | 29.5% of rivaroxaban, 74.1% of dabigatran, 57.6% of apixaban, and 71.0% of edoxaban groups received low dose NOAC. Others received standard dose NOAC. | NA |
| Seiffge DJ-2019 | 2,656 patients received standard dose of NOACs, 33.10% of rivaroxaban, 36.10% of dabigatran, 0.4% of edoxaban. 27.2% of apixaban | NA |
| Xian Y-2019 | 4,041 patients received standard dose of NOACs, 45.58% of rivaroxaban, 30.66% of dabigatran, 23.90% of apixaban. | 58-68 |
| Larsen TB-2014 | 2,398 patients received different dose of NOACs, 55.88% of low dose dabigatran, 44.12% of standard dose dabigatran. | NA |
| Komen JJ-2019 | 21,028 patients received standard dose of NOACs, 9.60% of rivaroxaban, 15.10% of dabigatran, 0.2% of Edoxaban, 75.2% of apixaban. | NA |
| Park J-2019 | 32,729 patients received standard dose of NOACs, 37.61% of rivaroxaban, 19.22% of dabigatran, 16.16% of Edoxaban, 27.00% of apixaban. | 49 |
| Coleman CI-2017 | 6.2% of rivaroxaban, 17.7% of dabigatran, 57.6% of apixaban, and 71.0% of edoxaban groups received low dose NOAC. Others received standard dose NOAC. | NA |
| Nielsen PB-2019 | NA | NA |
| Tsai C-2020 | 3,493 patients received standard dose of NOACs, 48.26% of rivaroxaban, 40.94% of dabigatran, 16.16% of Edoxaban, 10.79% of apixaban. | NA |
| Lee S-2020 | 63.3% of rivaroxaban, 74.1% of dabigatran, 57.6% of apixaban, and 71.0% of edoxaban groups received reduced dose NOAC. Others received standard dose NOAC. | NA |

NOACs=non-Vitamin K antagonist oral anticoagulants; VKAs=vitamin K antagonists;TTR=Time in the therapeutic range; NA=not available


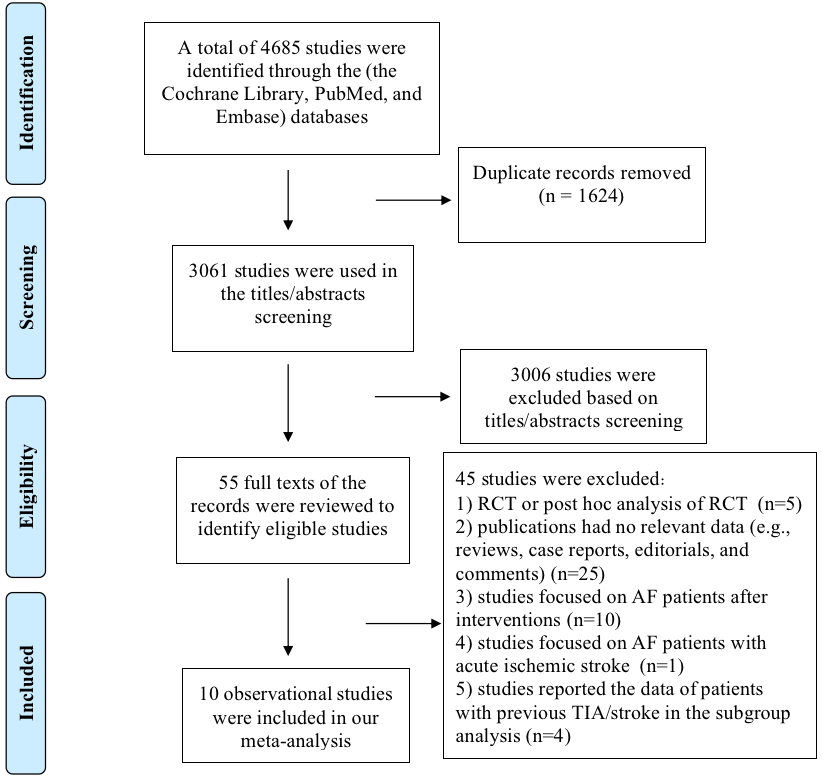


**Supplemental Figure 1. The literature retrieval process of this meta-analysis**
